# Surfactant application during extracorporeal membrane oxygenation improves lung volume and pulmonary mechanics in children with respiratory failure

**DOI:** 10.1186/cc3880

**Published:** 2005-10-25

**Authors:** Michael Hermon, Gudrun Burda, Christoph Male, Harald Boigner, Walter Ponhold, August Khoss, Wolfgang Strohmaier, Gerhard Trittenwein

**Affiliations:** 1 Professor of Pediatrics, Consultant in Pediatric Intensive Care Unit, Division of Neonatology and Pediatric Intensive Care, University Children's Hospital, Medical University of Vienna, Austria; 2 Consultant in Pediatric Intensive Care Unit, Division of Neonatology and Pediatric Intensive Care, University Children's Hospital, Medical University of Vienna, Austria; 3 Professor of Pediatrics, Consultant in Pediatric Cardiology, Division of Pediatric Cardiology, University Children's Hospital, Medical University of Vienna, Austria; 4 Fellow in Pediatric Intensive Care Unit, Division of Neonatology and Pediatric Intensive Care, University Children's Hospital, Medical University of Vienna, Austria; 5 Professor, Head of the Pediatric Radiology Department, Division of General Pediatrics and Pediatric Radiology, University Children's Hospital, Medical University of Vienna, Austria; 6 Consultant of Pediatric Radiology, Division of General Pediatrics and Pediatric Radiology, University Children's Hospital, Medical University of Vienna, Austria; 7 Professor of Biochemistry, Scientific Advisor, Ludwig Boltzmann Institute for Experimental and Clinical Traumatology, Vienna, Austria; 8 Professor of Pediatrics, Head of the Pediatric Intensive Care Unit, Division of Neonatology and Pediatric Intensive Care, University Children's Hospital, Medical University of Vienna, Austria

## Abstract

**Introduction:**

This study was performed to determine whether surfactant application during extracorporeal membrane oxygenation (ECMO) improves lung volume, pulmonary mechanics, and chest radiographic findings in children with respiratory failure or after cardiac surgery.

**Methods:**

This was a retrospective chart review study in a pediatric intensive care unit (PICU). Seven patients received surfactant before weaning from ECMO was started (group S). They were compared to six patients treated with ECMO who did not receive surfactant (group C). These control patients were matched based on age, weight, and underlying diagnosis. Demographic data, ventilator settings, tidal volume, compliance of respiratory system (calculated from tidal volume/(peak inspiratory pressure – positive end-expiratory pressure), and ECMO flow were extracted. Chest radiographs were scored by two blinded and independent radiologists. Changes over time were compared between groups by repeated-measures analysis of variance (time*group interaction). Values are given as percentages of baseline values.

**Results:**

The groups did not differ with regard to demographic data, duration of ECMO, ventilator settings, PICU and hospital days. After application of surfactant, mean tidal volume almost doubled in group S (from 100% before to 186.2%; *p* = 0.0053). No change was found in group C (100% versus 98.7%). Mean compliance increased significantly (*p* = 0.0067) in group S (from 100% to 176.1%) compared to group C (100% versus 97.6%). Radiographic scores tended to decrease in group S within 48 h following surfactant application. ECMO flow tended to decrease in group S within 10 h following surfactant application but not in group C. Mortality was not affected by treatment.

**Conclusion:**

Surfactant application may be of benefit in children with respiratory failure treated with ECMO, but these findings need confirmation from prospective studies.

## Introduction

Extracorporeal membrane oxygenation (ECMO) provides temporary extracorporeal life support for children with severe respiratory or cardiac failure. Since 1989, over 27,000 children have received ECMO with an overall survival rate of 76% [1]. Overall survival for children needing cardiac support is 58% [1]. ECMO therapy helps to reduce the barotrauma and high-inspired oxygen concentrations used in conventional mechanical ventilation. During ECMO, patients receive minimal respiratory support while the lungs are bypassed and allowed to heal. This technology has become safer and more efficient but complications may still occur, including mechanical complications in the extracorporeal circuit itself and complications of a patient's clinical status. The longer the duration of ECMO, the more complications will occur [2,3].

During the initial course of ECMO, chest radiographs show diffuse opacification corresponding to variable loss in lung volume, which leads to decreased lung compliance. When lung function improves, the lung volume re-expands with a concomitant increase in lung compliance and improved aeration as seen on chest radiographs [4]. The use of exogenous surfactant to reduce the duration of ECMO has previously been recommended by some authors [5,6]. The only reliable indicator so far to determine when to stop ECMO therapy is the return of adequate pulmonary and/or cardiac function. As oxygenation improves, tidal volume (VT) increases and chest radiographs reveal a reduction in pulmonary opacification, and less ECMO flow is required to maintain adequate arterial and mixed venous pO_2 _or saturation.

The objective of the current study was to assess whether surfactant application had an impact on VT, compliance of respiratory system (Crs), chest radiographic findings, and ECMO flow in children with respiratory failure or after cardiac surgery treated with ECMO.

## Materials and methods

### Patients

This study was designed as a retrospective review of all children (n = 49) treated with ECMO in our pediatric intensive care unit (PICU) between 1999 and 2001. ECMO entry criteria at our hospital include: weight greater than 2 kg body weight, maximal medical therapy consisting of a high fraction of inspired oxygen, and high pressure settings on the respirator, as well as an oxygenation index greater than 40.

Patients with acute respiratory failure were included, defined according to the criteria of the American European consensus conference on acute respiratory distress syndrome (ARDS) [7]. Patients with congenital heart disease who could not be weaned from the cardiopulmonary bypass after cardiac surgery were also included in the study.

Medical records of all ECMO patients were reviewed to determine whether the child received exogenous surfactant after initiation of ECMO. Seven patients received exogenous surfactant before starting the weaning procedure from ECMO (group S). The physician on duty decided on surfactant application individually. Of the patients who were treated with ECMO but did not receive surfactant at any time during their hospital course, six patients matched on age, weight, and underlying diagnosis, but not on the basis of 'severity of disease', were chosen as controls (group C). Patients' medical records were reviewed for the following information: demographic data, ventilator settings (peak inspiratory pressure (PIP), positive end-expiratory pressure (PEEP), fraction of inspired oxygen (FiO2), respiratory rate, VT), and ECMO flow. Crs was estimated by the ratio VT/(PIP – PEEP) from a single expiratory tidal volume. This was performed without disconnecting the patient from the ventilator, the only limitation being that a single VT and pressure difference was used to calculate compliance. The time points for this information determined for group S patients were: before surfactant application (baseline); and 4 h and 10 h after surfactant application. Time points for group C patients were: baseline (mid-time of ECMO course) and 4 h and 10 h after baseline. These time points were chosen to roughly correspond to the course in group S patients, who received their surfactant approximately at the mid-time of the ECMO course.

Chest radiographs were evaluated by two independent pediatric radiologists blinded to treatment groups. The respiratory distress syndrome severity scoring system devised by Edwards *et al*. [8] with a score range from 4 to 20 was used. This score evaluates the following criteria: degree of aeration and pulmonary opacification, presence of air bronchograms, and cardiac and diaphragmatic silhouette definition.

Chest radiographs for group S were obtained before surfactant application (baseline), 24 and 48 h thereafter, and for group C at baseline (mid-time of the ECMO course), and 24 and 48 h thereafter.

Children in group S received 50 to 80 mg/kg body weight porcine surfactant (Curosurf^®^, Chiesi, Italy) as an intratracheal bolus. The children were handbag ventilated during surfactant application with 100% oxygen using peak pressures and respiratory rates that approximated previous ventilator settings on ECMO. The whole application procedure lasted about 2 minutes. Endotracheal suctioning was not performed within 4 h after surfactant application.

All patients were ventilated with a time cycled, pressure controlled ventilator (Babylog 8000, Dräger, Lübeck, Germany). Hemodynamic variables were monitored online by using a pressure transducer and ECG electrodes and displayed on a monitor system (Hewlett-Packard, Model 68 S, Palo Alto, CA, USA). All patients were treated with midazolam and fentanyl analgesia and received pancuronium bromide for relaxation.

Veno-arterial ECMO was initiated using neck cannulation in two children and through median sternotomy in four children. In these latter children, the sternum was left open with primary skin closure. For veno-venous ECMO (n = 7), we used right internal jugular vein and right femoral vein access. The heparin-coated ECMO circuit consisted of a Biomedicus BP 50 centrifugal pump head (Medtronic Inc., Anaheim, CA, USA) and a Quadrox D (Jostra, Hirrlingen, Germany) oxygenator.

### Statistical analysis

Statistical analysis was performed with commercially available computer software packages (Minitab version 13.1(Minitab Inc., Lebanon, PA, USA), SAS/STAT, version 8.2 (SAS Institute Inc., Cary, NC, USA)). Demographic variables were compared between groups using Wilcoxon sign rank test or Fisher's exact test. For outcome variables (VT, Crs, respiratory distress syndrome severity score (RDS), ECMO flow), baseline differences between groups were assessed using Student's *t* test. Changes over time were compared between groups by repeated-measures analysis of variance (time*group interaction). Values are given as percentage of baseline values. All tests were two-sided. Significance for all comparisons was accepted at *p* < 0.05.

The study was exempted from review by the Ethics Committee of the Medical University of Vienna and from the requirement for informed consent because it involved the examination of existing data and documents.

## Results

Between 1999 and 2001, 857 children were admitted at our PICU. Of these children, 49 were treated with ECMO (5.8% of all admitted children): 17 children (35% of all ECMO patients) had ARDS, 23 children (46%) had congenital heart disease, and 9 children (18%) had another diagnosis. Seven children were treated with surfactant during ECMO (group S; 14% of all ECMO patients). Six children who did not receive surfactant were matched as a control group (group C). Demographic data of both groups are listed in Table 1. Groups were matched based on age, weight, and underlying diagnosis. There were no significant differences between the two groups regarding all demographic data. The most common diagnosis in both groups was ARDS. Comparison of the two groups for duration of ECMO, PICU, ventilator and hospital days revealed no significant differences.

**Figure 1:**
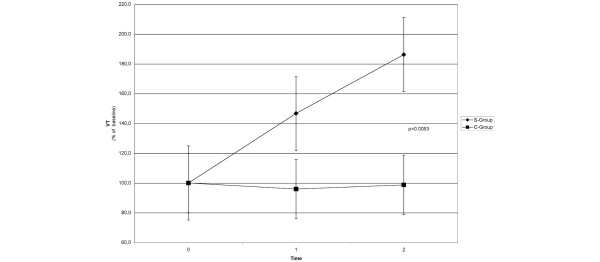
Tidal volumes (VT) of the surfactant group (group S) and control group (group C). The X-axis represents the time points before (baseline) and 4 and 10 h after surfactant application for group S. For group C, the time points are baseline (mid-time of the ECMO course) and 4 and 10 h thereafter. The Y-axis represents values of VT as percentages of baseline values. VT showed a significant increase over time in group S compared to group C (repeated-measures, analysis of variance, group*time interaction, *p* = 0.0053).

**Figure 2:**
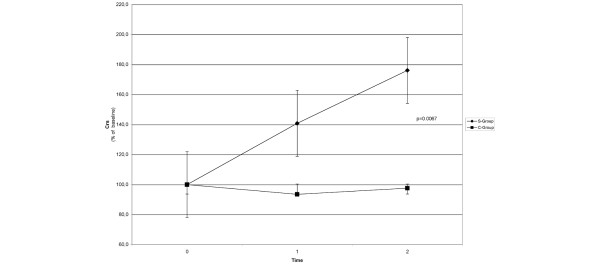
Compliance of respiratory system (Crs) calculated from the ratio tidal volume/(peak inspiratory pressure – positive end-expiratory pressure) for the surfactant group (group S) and control group (group C). The X-axis represents the time points before (baseline) and 4 and 10 h after surfactant application for group S. For group C, the time points are baseline (mid-time of the ECMO course) and 4 and 10 h thereafter. The Y-axis represents values of Crs as percentages of baseline values. Crs showed a significant increase over time in group S compared to group C (repeated-measures, analysis of variance, group*time interaction, *p* = 0.0067)

The measured variables VT, Crs and RDS showed moderate differences between groups at baseline (*p* values not significant). Independent of baseline differences, however, these variables showed significant changes over time as assessed by repeated measures analysis of variance. All values are given as changes in percentage of baseline values. Mean VT improved significantly over time in group S (100% at baseline versus 186.2% at 10 h after surfactant application) compared to group C (100% versus 98.7%; *p* = 0.0053) (Figure 1). Similarly, mean Crs values increased significantly over time in group S (100% before versus 176.1% at 10 h after surfactant application) compared to group C (100% versus 97.6%; *p* = 0.0067) (Figure 2).

**Figure 3:**
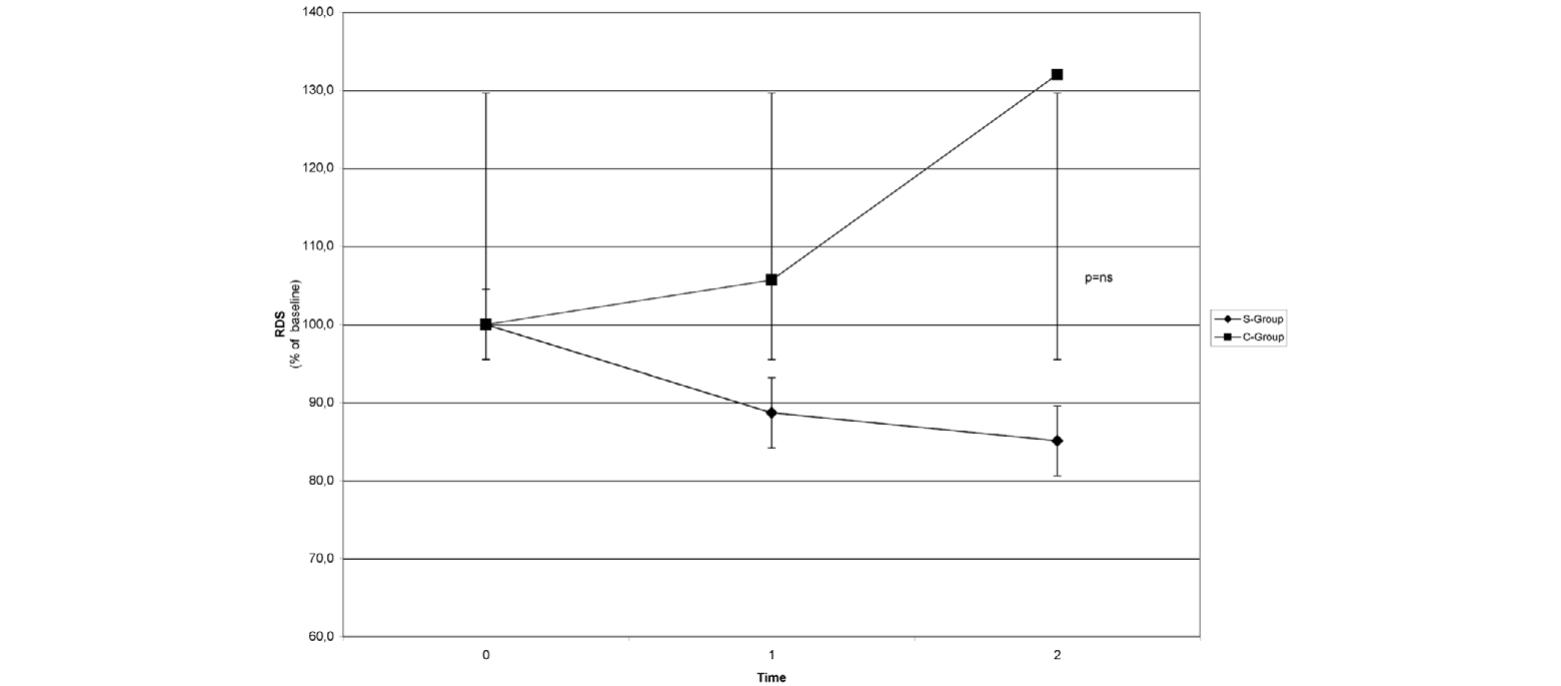
Respiratory distress syndrome severity score (RDS) for the surfactant group (group S) and control group (group C). The X-axis represents time points when radiographs were obtained and scored (baseline (before surfactant application) and 24 and 48 h thereafter) for group S, and for group C at baseline (mid-time of ECMO-course) and 24 and 48 h thereafter. The Y-axis represents the radiographic RDS score.

**Figure 4:**
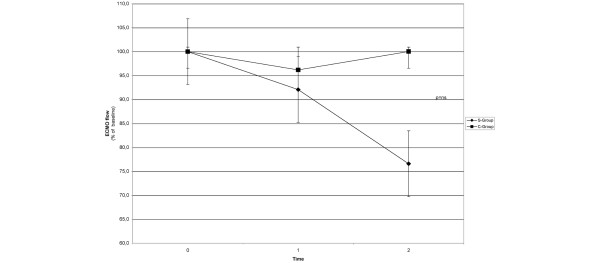
ECMO flow for the surfactant group (group S) and control group (group C). The X-axis represents the time points before (baseline) and 4 and 10 h after surfactant application for group S. For group C, the time points are baseline (mid-time of the ECMO course) and 4 and 10 h thereafter. The Y-axis represents ECMO flow as percentages of baseline values.

Radiographic scores are shown in Figure 3. Mean RDS values of group S improved moderately (100% at baseline versus 61.1% at 48 h after surfactant application). In group C, mean RDS values did not change within 24 h, but then increased to 132%, evidence of a mild aggravation (*p* = 0.14). Mean ECMO flow (l/minute) decreased over time in group S (100% at baseline versus 76.6% at 10 h after surfactant application). In group C, ECMO flow did not change over the measured time points (100% versus 100.03%, *p* = 0.18) (Figure 4). Survival in group S was 29% and in group C 50% (*p* = 0.59).

## Discussion

We found that surfactant application in children with respiratory failure treated with ECMO was associated with improved lung volume and pulmonary mechanics within 10 h. Tidal volume and Crs improved significantly in the surfactant group compared to the control group over the course of time. In addition, chest radiograph scores showed a trend to improvement in the surfactant group but not in the control group. Moreover, ECMO flow tended to decrease 10 h after surfactant application. However, there was no significant difference in overall outcome.

To our knowledge there have been only few previous studies describing surfactant therapy during ECMO [5,6]. These studies reported on term neonates, however, and not on infants and children as in our present study. This study was performed as a retrospective case control study. The decision for treatment with surfactant during ECMO was made by the physician on duty on an individual basis. Patients of group C were matched based on age, weight and underlying diagnosis, but not according to the severity of disease. This explains why baseline values differed between the two groups, but not significantly. However, changes over time expressed as percentage of baseline values showed significant differences between both groups.

ECMO remains a useful technique in the management of children with respiratory failure. The optimal timing for placing children on ECMO is still difficult to determine. Greenspan *et al*. [9] suggested that delaying ECMO therapy might increase the risk of lung injury, particularly to the airway. New treatment strategies and ventilator techniques for respiratory failure were introduced in the past decade with the implication of fewer requirements for ECMO. These new treatment strategies, such as inhaled nitric oxide, high frequency ventilation and surfactant replacement, may be used as co-therapy with ECMO in order to shorten ECMO runs and thereby reduce complications with ECMO [3].

Multiple causes of surfactant deficiency exist in children requiring ECMO, making surfactant replacement a treatment option. Hyperventilation and hyperoxia, often required for the patient with severe respiratory failure and persistent pulmonary hypertension, can lead to barotraumas and oxygen toxicity [10]. Surfactant function and pulmonary mechanics are impaired by the influx of protein-rich fluid and blood into the alveolar space [11-13]. Pulmonary edema is usually present for the first 48 to 72 h after initiation of ECMO as assessed by chest radiographs [13]. Prevention of this initial course may be clinically important to reduce ECMO duration and to avoid high ventilation requirements during the weaning period from ECMO therapy and after removing the patient from bypass.

Some investigators have previously reported improved pulmonary mechanics and decreased duration of ECMO in neonates who had received multiple doses of surfactant while on extracorporeal bypass [5,6]. Our patients were infants and children (mean age of 6 months) and received only one dose of surfactant. The average duration of ECMO in neonates is 120 h [14]. Our patients in both groups remained on ECMO approximately 215 h. We agree with Green and coworkers [14] who argued that for the special case of veno-arterial ECMO, it seems unlikely that ECMO has any direct therapeutic effect in acute lung-injured patients separate from its support of gas exchange. Therefore, the duration of ECMO must be long enough to allow substantial lung repair to occur. The duration of ECMO correlates with measurable indicators of severe pulmonary disease, such as PIP and prolonged mechanical ventilation before ECMO.

ECMO is reserved in most centers only for patients for whom the likelihood of survival with the continuation of conventional therapy appears remote. In that respect, the overall survival of our patients (mean of both groups 40%) appears encouraging.

The assessment of VT and Crs may offer the clinician a possibility of a more objective evaluation of pulmonary status and the recovery from lung injury compared with the conventional assessment by chest radiographs and blood gases [15]. Chest radiograph findings lag behind the clinical and physiological recovery of the lung. Blood gases during ECMO mainly reflect gas exchange in the membrane lung (Oxygenator) of the ECMO circuit and only in a small amount reflect gas exchange in a patient's lungs. Compliance increased significantly in the surfactant group after surfactant application, indicating alveolar recruitment of the lung as pressure difference (PIP – PEEP levels) was almost unchanged over the different time points measured. Reiterer *et al*. [16] and previous investigators [5,17-20] have described improvement of compliance during ECMO and especially in combination with surfactant treatment. They mentioned that Crs, functional residual capacity and VT improved significantly, and each of these parameters correlated with successful weaning from ECMO. The combination of functional residual capacity and Crs was the best predictor for successful weaning from ECMO [17]. The decision of when to stop ECMO is based upon the return of adequate pulmonary and cardiac function to support vital organs and permit subsequent recovery [21].

## Conclusion

We found that surfactant replacement during ECMO in children with respiratory failure improved lung volume; pulmonary mechanics and measurement of these parameters may assist weaning from ECMO. ECMO reduces the need for high levels of respiratory support and it might be reasonable not to delay the initiation of it. The central question whether surfactant application during ECMO may improve outcome also in terms of higher survival has to be investigated in prospective controlled clinical trials.

## Key messages

• 	ECMO provides temporary life support for children with severe respiratory or cardiac failure. Although ECMO has become safer and more efficient, complications are still a threat.

• 	Surfactant replacement during ECMO in children with respiratory failure improved lung mechanics and supported tolerance to and assisted weaning from ECMO.

## Abbreviations

ARDS = acute respiratory distress syndrome; Crs = compliance of respiratory system; ECMO = extracorporeal membrane oxygenation; PEEP = positive end-expiratory pressure; PICU = pediatric intensive care unit; PIP = peak inspiratory pressure; RDS = respiratory distress syndrome severity score; VT = tidal volume.

## Competing interests

The authors declare that they have no competing interests.

## Authors' contributions

MH conceived the study, participated in the design and execution of the study, the analysis of data and writing of the manuscript. CM performed the statistical analysis and interpretation of the data. GB and HB performed data collection. WP and AK performed the radiology analysis of chest radiographs. WS participated in the study design, interpretation of results and writing of the manuscript. GT supervised the study and is the ECMO program director. All authors read and approved the final manuscript

## Figures and Tables

**Table 1:** Demographic and clinical data of extracorporeal membrane oxygenation patients (n = 49) between 1999 and 2001

	Group S (surfactant; n = 7)	Group C (control; n = 6)	Remaining ECMO patients (n = 36)
Gender (male/female)	5/2	6/0	25/11
Age (months)^a^	5.1 ± 8.5	7.5 ± 9.7	11 ± 25.1
Weight (kg)^a^	5.1 ± 3.5	6.0 ± 4.1	6.3 ± 7.3
Diagnosis (ARDS/CHD)^a^	5/2 (71%/29%)	4/2 (67%/33%)	8/19 (22%/53%)
ECMO (days)	9 ± 7.9	9 ± 6.2	6.7 ± 4.9
ECMO (hours)	217.4 ± 198	214.8 ± 152	154.2 ± 117.2
PICU (days)	24.5 ± 19.2	19.5 ± 4.3	18.3 ± 14.1
Ventilator (days)	23.5 ± 17.7	17.5 ± 5.1	16.2 ± 13.4
Hospital (days)	29.5 ± 18.3	23.2 ± 8.8	22.3 ± 16
Survival (y/n)	2/5	3/3	16/20 (44%/56%)

^a^Matching variables. Data are presented as mean ± standard error of the mean. There were no significant differences between the two groups regarding all demographic data. ARDS, acute respiratory distress syndrome; CHD, congenital heart disease; ECMO, extracorporeal membrane oxygenation; PICU, pediatric intensive care unit.
